# An overview of the expert consensus on the mental health treatment and services for major psychiatric disorders during COVID-19 outbreak: China's experiences

**DOI:** 10.7150/ijbs.47419

**Published:** 2020-05-25

**Authors:** Yu-Tao Xiang, Na Zhao, Yan-Jie Zhao, Zihan Liu, Qinge Zhang, Yuan Feng, Xiao-Na Yan, Teris Cheung, Chee H. Ng

**Affiliations:** 1 Unit of Psychiatry, Institute of Translational Medicine, Faculty of Health Sciences, University of Macau, Macao SAR, China.; 2 Center for Cognition and Brain Sciences, University of Macau, Macao SAR, China.; 3 The National Clinical Research Center for Mental Disorders & Beijing Key Laboratory of Mental Disorders, Beijing Anding Hospital & the Advanced Innovation Center for Human Brain Protection, Capital Medical University, Beijing, China.; 4 Xiamen Xianyue Hospital, Fujian province, China.; 5 School of Nursing, Hong Kong Polytechnic University, Hong Kong SAR, China.; 6 Department of Psychiatry, The Melbourne Clinic and St Vincent's Hospital, University of Melbourne, Richmond, Victoria, Australia.

**Keywords:** Expert consensus, major psychiatric disorders, online mental health service, COVID-19, China

## Abstract

The 2019 novel coronavirus disease (COVID-19) epidemic in China has presented substantial challenges to traditional forms of mental health service delivery. This review summarizes the expert consensus on the mental health treatment and services for severe psychiatric disorders during the COVID-19 outbreak developed by the Chinese Society of Psychiatry and other academic associations. The Expert Recommendations on Managing Patients with Mental Disorders during a Serious Infectious Disease Outbreak (COVID-19) outline the appropriate measures for psychiatric hospitals or psychiatric units in general hospitals, including the delivery of outpatient, inpatient, and community mental health services. The Expert Recommendations on Internet and Telehealth in Psychiatry during Major Public Health Crises (COVID-19) describe the assessment and treatment issues of internet-based mental health services during the COVID-19 outbreak. The expert consensus recommendations provide guidance for mental health professionals in managing psychiatric services during the COVID-19 outbreak in China. The experiences from China in addressing the challenges in the management of major psychiatric disorders may be useful and relevant to other countries who are combating the COVID-19 pandemic.

## Introduction

At the end of 2019, the novel coronavirus disease (COVID-19) was first identified in Wuhan, Hubei province, China, before it became a pandemic that spread to more than 200 countries. As of May 7, 2020, there have been 83,974 infected cases in China and 3,775,667 cases globally 1. Among the population most at risk, patients with major psychiatric disorders are particularly susceptible to the novel infection.

There are several factors which contribute to this vulnerability. Psychiatric patients treated in mental hospitals often experience crowded living conditions compared to their counterparts in general hospitals. They may lack ability to practice effective infection control measures to protect themselves due to their poor mental health. Further, they are often required to participate in group therapy activities and share common bathroom and dining halls and toilets, all of which increase the risk of transmission. In addition, due to unhealthy lifestyle, poor self-care ability, and suboptimal health status associated with psychiatric disorders, they are more vulnerable to COVID-19 than those without psychiatric disorder 2.

The community mental health services are not well established in many regions in China, and therefore, follow-up maintenance treatments are mostly provided by outpatient clinics in large psychiatric hospitals in urban areas 3,4. To obtain their maintenance medications regularly, most patients with severe psychiatric disorders are required to visit hospital outpatient clinics, which increases their exposure to nosocomial infections such as the COVID-19. For instance, more than 300 patients with severe psychiatric disorders were infected with the COVID-19 in early stage of the COVID-19 outbreak in China 5. Nevertheless, information on the number of infected mental health professionals in China was not released by the health authorities.

During the COVID-19 outbreak, the delivery of traditional face-to-face mental health services has been severely restricted due to the high risk of virus transmission. Hence, alternative services utilizing digital technology such as the use of smartphone, Internet and social communication media (e.g., WeChat and Weibo), and online mental health services, have been widely implemented in China 6. In order to provide timely guidance for frontline health professionals during the COVID-19 outbreak, several specific expert consensuses for mental health services during the COVID-19 outbreak have been disseminated in China 7. Of note, some of these recommendations are consistent with the guidelines published by the Inter Agency Standing Committee (IASC) Guidelines for Mental Health and Psychosocial Support in Emergency Settings 8.

The experiences from China in implementing these guidelines to tackle the mental health challenges may benefit other countries that are still combating the COVID-19 pandemic. However, as these guidelines were developed and published in Chinese, they are not readily accessible by the international readership. For these reasons, we present here an overview of the expert consensus on the management of patients with major psychiatric disorders as well as the online mental health services during the COVID-19 outbreak in China.

## Expert Recommendations on Managing Patients with Mental Disorders during COVID-19 9

On February 18, 2020, the State Council of China released a notification on “Strengthening the Treatment and Management of Patients with Severe Psychiatric Disorders during the COVID-2019 Outbreak” 10, to guide mental health institutions in preventing hospital-acquired infection during the treatment and care of patients with severe psychiatric disorders.

Due to the risk factors faced by patients with severe psychiatric disorders mentioned previously as well as the difficulty in maintaining social distancing in most psychiatric hospitals or psychiatric wards of general hospitals in China, the Chinese Society of Psychiatry released "the Expert Recommendations on Management Strategies for Patients with Mental Disorders to Prevent and Control the Outbreak of a Serious Infectious Disease (COVID-19)" 9. The membership of the expert group is shown in the Supplementary tables. The process of establishing a consensus was as follows: a panel of key experts prepared the draft, which was sent to all members of the expert group, and based on the feedback the final version of the guideline was established.

### General measures for psychiatric hospitals or psychiatric units of general hospitals

During the COVID-19 epidemic, psychiatric hospitals should regularly disinfect or clean the hospital grounds, reduce number of the entrances and exits into the hospital, and set up temperature measurement screening at the hospital entrance. To reduce the risk of community transmission within the hospital, psychiatric hospitals must take necessary measures to reduce the number of outpatient visits, set up isolation observation wards for newly admitted patients, shorten hospital length of stay, and suspend all family visits. They must regularly provide training on COVID-19 diagnosis and treatment for hospital staff. In particular, all hospital staff must strictly observe the relevant regulations of the local government in terms of the need for home isolation or centralized medical testing and observation to address any symptom development of COVID-19 in patients.

### Measures for outpatient departments

If possible, psychiatric outpatient clinic of general hospital should be situated away from emergency departments, fever clinics, respiratory department and other departments where there is a high risk of infection. Outpatient clinics should be well ventilated, and personal protective equipment (PPE), such as hand sanitizer, paper towel, masks, protective caps, and gloves, should be adequately supplied.

Single entrance into outpatient department is recommended, and all patients and their guardians must wear face masks and have their temperatures checked on entry. Only one guardian is allowed to accompany each patient. Patients and guardians with fever or respiratory symptoms are strictly prohibited from entering the outpatient department.

At the screening (triage) station, patients with fever and/or other clinical symptoms or related epidemiological history of COVID-19 should be managed according to the relevant local regulations, such as being transferred to a local designated medical facility and fever clinics. Patients with severe psychiatric disorders and fever and/or other clinical symptoms or related epidemiological history should be treated by the on-duty psychiatrists with PPE in designated isolation rooms or ward in the psychiatric hospital.

During the psychiatric consultation, the treating psychiatrist should not only assess for psychiatric disorders, but also confirm if there is any history of fever, respiratory symptoms and epidemiological history of COVID-19. If any positive history is identified, they need to record and report to the relevant administrative departments as required and initiate relevant transfer procedures.

The interval between follow-up outpatient appointments should be adjusted accordingly, and remote delivery of treatment, such as online services, should be encouraged. For stable patients, extension of appointment intervals of up to 3 months could be considered. Medication adherence should be reinforced and psychoeducation for both patients and guardians should be provided. In the event of any worsening of mental state, instruction on how to seek professional help should be given. Specialised treatments, such as psychological evaluation, psychological counseling, psychotherapy, rehabilitation treatment and electroconvulsive therapy (ECT), should be appropriately limited during the epidemic, and even suspended, if necessary, to reduce the risk of transmission. Similarly, the frequency of regular physical examination and adverse drug effects monitoring should be decreased where appropriate.

As far as possible, telehealth, telephone and other online mental health services should be encouraged. Psychotropic medications could be organized and delivered to patients in need through third-party delivery services. Patients who need on-site treatment or hospitalization are recommended to seek help directly from psychiatric hospitals close to their residential home.

### Measures for inpatient departments

Similarly, having a single entrance to the inpatient department with a requirement for wearing mask and temperature screening for all persons entering is important. If possible, isolation wards should be set up with sufficient PPE supply in hospitals for observation of all newly admitted patients.

The admission rate into the inpatient departments should be reduced by strengthening the treatment program in the outpatient departments and community mental health services during the COVID-19 epidemic. For patients who need to be hospitalized due to severe excitement, agitation, impulsive behaviour, suicidal behaviour or deliberate self-harm, but do not have COVID-19-related epidemiological history and clinical symptoms, it is recommended that screening with body temperature, routine blood check-up, chest Computed Tomography or X-ray and other necessary examinations are performed, to exclude the possibility of COVID-19. It is suggested that 14 days observation in isolation wards should be mandatory before transferring new patients to the general wards. Psychiatric patients with suspected or confirmed COVID-19 infection must be referred to the local designated hospitals, while psychiatric hospitals should also provide consultation services to the local designated hospitals.

In the management of hospitalized patients, the diagnosis and treatment plans should be expedited, and the length of stay should be shortened as much as possible to reduce the number of inpatients at any one time. In principle, examination and treatment of hospitalized patients should be performed within the same inpatient department. Inpatients should be restricted to enter other departments to avoid the risk of cross infection between patients in different departments. If certain equipment or treatment facilities are shared between inpatients and outpatients, different time slots should be adopted, and disinfection and cleaning must be strictly enforced between sessions.

All types of leave from hospital (e.g., day leave and home leave) and routine family visits should be suspended during the epidemic. If patients require treatment for physical illnesses, or have other specific reasons to leave or be transferred to another hospital, they should be discharged from the psychiatric hospital. Telecare, WeChat or remote social media could be used to facilitate communication between patients and their families, and hospital staff can facilitate these logistics for hospitalized patients.

### Measures for community mental health services

For stable patients with severe psychiatric disorders who reside in the community during the COVID-19 epidemic, the use of telehealth, telephone and other online services for the ongoing monitoring and treatment requirements should be promoted. These strategies could be used in monitoring patients' mental state, delivering psychoeducation for family members, providing medication management as well as ensuring epidemic prevention and control. Frequency and length of face-to-face visits should be proportionally reduced where possible. If face-to-face assessment is deemed necessary, mental health professionals should maintain social distancing, proper handwashing and adherence to other self-protective/ hygienic measures.

## Expert Recommendations on Internet and Telehealth in Psychiatry during Major Public Health Crises (COVID-19) 11

In any public health crisis, timely delivery of health assessment and professional treatment, without increasing the risk of community transmission or pressure on hospital service system, is essential. As such, the General Office of the National Health Commission of China released the notification on “Internet-Based Assessment, Treatment and Counseling Services in Healthcare Delivery during the COVID-19 Epidemic” 12, to promote the use of "Internet + Medical Care" model during the COVID-19 epidemic. The membership of the expert group is shown in the Supplementary tables. Similarly, a panel of key experts prepared the draft, which was sent to all members of the expert group, and based on the feedback the final version of the guideline was established.

While patients with physical disorders commonly require physical examinations, investigations and procedures in medical institutions, those with mental disorders, on the other hand, often need mental state examinations, long-term maintenance medications and psychotherapies. Hence their mental health needs could be met more readily via telehealth or internet-based mental health services. By minimizing face to face contact through these alternative forms of services, patients with mental health disorders could reduce the risk of cross infection, shorten the delay in help-seeking, and have better access to mental health treatment and services.

To ensure the quality of internet-based mental health services, the Chinese Society of Psychiatry, the Chinese Psychiatrist Association, the Mental Health Center of the Chinese Center for Disease Control and Prevention jointly developed "The Expert Recommendations on Internet and Telehealth in Psychiatry during Major Public Health Crises (COVID-19)".

Internet-based mental health services should register with the local health authorities, and follow their relevant regulations and policies. Ethical principles, scope and types of online medical services, fee structure, health insurance, referral standards, and legal issues related to online service disputes need to be considered according to the local legal and sociocultural contexts.

For continuity of care, as far as possible, the online doctor should be the treating doctor of the patient with mental disorder. If this is not possible, another doctor with similar clinical expertise from the same hospital delivering the treatment can also provide the online follow-up consultation. Similarly, the treating doctor should give priority to their own patients to follow-up their online treatment. For patients who are treated by other doctors not familiar with their history, they should be informed of the potential shortcomings and risk of online medical services. The online medical services can only proceed after obtaining the patient's informed consent. In assessing new patients who have not been assessed previously, doctors should not establish diagnosis or prescribe treatment since reliable assessment and examination cannot be conducted online. At most, doctors can provide some general counseling service as well as advice about further on-site diagnosis and treatment needed. The limits of online services should be clearly explained to the patients.

As the core component of online mental health services, the online consultation can employ graphic/text, telephone/voice, and telehealth/video modalities, of which, graphic/text counseling is the most widely used. The telephone/voice and telehealth/video consultation appointment should be arranged prior to the consultation. The consultation by the doctor should be conducted in a quiet location without any interruption. In the context of the COVID-19 epidemic, information about the epidemic in the patients' location, infection of their family or relatives, and potential psychological impact of related issues on patients should be assessed as these factors may influence their mental status.

To facilitate online assessment, some self-reported validated scales, such as the 15 items of Patient Health Questionnaire, Self-Rating Depression Scale, Self-Rating Anxiety Scale, and Pittsburgh Sleep Quality Index, could be considered. If further investigations are needed, patients should be advised to attend on-site assessment.

For online treatment delivery, psychological counseling or other psychotherapies should be conducted according to the relevant local guidelines. One of the key goals is to provide psychological crisis services for people affected by the COVID-19 epidemic, such as emotional counseling, psychosocial support and crisis intervention. In terms of medication prescriptions, these can only be provided online for clinically stable patients requiring long-term maintenance treatment. It is recommended that the amount of medication prescribed should not exceed 1 month and patients should be informed about the potential risk of medication, side effects and their management. For the follow up of patients who are not fully stable, it is recommended that the amount of medication prescribed should not exceed more than 2 weeks.

Online referral system could be established to improve efficiency of referral to the specialist psychiatric team. All online patients can be first screened by medical assistants and appropriate patients are first referred to junior doctors in the first instance. Subsequently, if the cases are complex, they can be promptly referred to senior consultants or other doctors with relevant expertise.

Finally, a national teleconsultation platform with a multidisciplinary team could be established to provide secondary consultation services delivered online for challenging issues, such as clinical difficulties faced by primary mental health services, or when psychiatric support and consultation are needed in general hospitals. Should any of the patients treated online require urgent on-site assessment or hospitalization, they should be appropriately referred to the nearest psychiatric hospital as soon as possible.

## Conclusion

The expert consensus recommendations provide guidance for mental health professionals in managing psychiatric services during the COVID-19 outbreak in China. Traditional forms of mental health services may need to be replaced or supplemented with online mental health services during the COVID-19 epidemic. The experiences from China in addressing the challenges in the management of major psychiatric disorders may be useful and relevant to other countries that are combating the COVID-19 pandemic. However, certain methodological issues should be noted. The processes and methodologies in developing these guidelines were not outlined in detail. In addition, the expert consensus recommendations were developed specifically for mental health professionals in China. Further, if the relevant guidelines are adapted to other countries, local sociocultural and economic factors should be considered, such as the accessibility of internet services for telehealth in psychiatry.

## Supplementary Material

Supplementary tables.
