# Supplementary material for: An overview of the expert consensus on the mental health treatment and services for major psychiatric disorders during COVID-19 outbreak: China's experiences
Source: Int J Biol Sci. 2020 May 25;16(13):2265–70. doi: 10.7150/ijbs.47419 (PMC7378640; doi:10.7150/ijbs.47419)
Supplement: Supplementary file 1 — Supplementary tables. [file ijbsv16p2265s1.pdf]

Supplementary tables

Membership of the Group for the Expert Recommendations on Managing Patients with Mental Disorders during COVID-19 [9]

Institute of Mental Health, The Second Xiangya Hospital of Central South University	Xilong Cui<br>Lingjiang Li<br>Xiaoping Wang<br>Jingping Zhao
Mental Health Center of Shanghai Jiao Tong University School of Medicine	Yiru Fang<br>Kaida Jiang<br>Bin Xie<br>Yifeng Xu<br>Mingyuan Zhang<br>Min Zhao
The First Affiliated Hospital of Chongqing Medical University	Li Kuang
West China Hospital of Sichuan University	Tao Li
Anhui Mental Health Center	Xiaosi Li
Wuhan Mental Health Center	Yi Li
Beijing Anding Hospital, Capital Medical University	Zhanjiang Li
The Sixth People's Hospital of Hebei Province	Keqing Li
Hubei Jingzhou Psychiatric Hospital	Bo Liu
Shenzhen Kangning Hospital	Tiebang Liu
The Sixth Hospital of Peking University	Lin Lu<br>Tianmei Si<br>Xin Yu
The First Affiliated Hospital of Xi 'An Jiaotong University	Xiancang Ma
Huashan Hospital of Fudan University	Shenxun Shi
The First Affiliated Hospital of China Medical University	Yanqing Tang
People's Hospital of Wuhan University	Gaohua Wang
Beijing Huilongguan Hospital	Shaoli Wang
Shanghai Hongkou Mental Health Center	Zuowei Wang
The First Affiliated Hospital of Kunming Medical University	Xiufeng Xu
The Affiliated Cancer Hospital of Chinese Academy of Sciences University	Enyan Yu
The General Hospital of Tianjin Medical University	Jianli Yang
Brain Hospital of Nanjing Medical University	Ning Zhang
The People's Hospital of Xinjiang Uygur Autonomous Region	Shaohong Zou

Membership of the Group for the Expert Recommendations on Internet and Telehealth in Psychiatry during Major Public Health Crises (COVID-19) [11]

The First Affiliated Hospital of Harbin Medical University	Jian Hu
Guangdong Provincial People's Hospital	Fujun Jia
Tianjin Anding Hospital	Jie Li
The Second Xiangya Hospital of Central South University	Lingjiang Li<br>Xiaoping Wang
West China Hospital of Sichuan University	Tao Li
The Sixth People's Hospital of Hebei Province	Keqing Li
Shenzhen Kangning Hospital	Tiebang Liu
The People's Hospital of Wuhan University	Zhongchun Liu<br>Gaohua Wang
The Sixth Hospital of Peking University	Lin Lu<br>Zhaojun Ni<br>Hongqiang Sun<br>Xin Yu
Tongji Hospital of Tongji University	Zheng Lu
The First Affiliated Hospital of Xi 'An Jiaotong University	Xiancang Ma
Brain Hospital of Guangzhou Medical University	Yuping Ning
The Seventh People's Hospital of Hangzhou	Hongjing Mao
Huashan Hospital of Fudan University	Shenxun Shi
Xijing Hospital of Air Force Medical University	Qingrong Tan
Sichuan Zigong Mental Health Center	Youguo Tan
The First Affiliated Hospital of China Medical University	Yanqing Tang
Beijing Anding Hospital of Capital Medical University	Gang Wang
Shandong Mental Health Center	Ruzhan Wang
Xiamen Mental Health Center	Wenqiang Wang
Shanghai Mental Health Center	Zhen Wang<br>Yifeng Xu
Peking Union Medical University Hospital	Jing Wei
The First Affiliated Hospital of Zhejiang University	Yi Xu
The First Affiliated Hospital of Kunming Medical University	Xiufeng Xu
Beijing Huilongguan Hospital	Fude Yang
The General Hospital of Tianjin Medical University	Jianli Yang
Brain Hospital of Nanjing Medical University	Ning Zhang
Dongfang Hospital of Tongji University	Xudong Zhao
The Second People's Hospital of Guizhou Province	Cao Zhou
